# 122 ”Chi si muove, si ama!” a multi-stakeholder and multi-disciplinary sub-national campaign to promote active lifestyle and physical activity in Italy

**DOI:** 10.1093/eurpub/ckae114.126

**Published:** 2024-09-26

**Authors:** Marco Bigica, Silvano Zanuso, Federica Alberti

**Affiliations:** Wellness Foundation, Cesena, Italy; Wellness Foundation, Cesena, Italy; Technogym, Cesena, Italy; Wellness Foundation, Cesena, Italy

## Abstract

**Purpose:**

Physical activity is crucial for longevity, yet many adults have a sedentary lifestyle which increases the risks of non-communicable diseases (NCDs) such as diabetes, hypertension, and cancer. National and local institutions must prioritize physical activity promotion to mitigate NCDs incidence, enhance citizens’ well-being, and reduce healthcare expenditures. “Chi si muove, si ama!” (“Moving is loving”) is an innovative campaign in Emilia-Romagna, Italy, targeting sedentary adults aged 40-60. It employs unconventional communication and engages public, private, and third-sector stakeholders including general practitioners, pharmacies, sports centers, gyms, and walking groups. This collaborative effort represents a holistic approach to health promotion, offering a coordinated health-promotion message and diverse avenues to increase physical activity levels.

**Project description:**

This project was developed as a collaboration between local and regional health authorities, professional associations of GPs and pharmacists, sports centres, gyms and health-promoting gyms, and walking groups. The project is coordinated by the Wellness Foundation.

The campaign was launched at the end of 2023 and is ongoing. It is based on the tailored contribution from various stakeholders: GPs offer personalized advice and educational material on physical activity; pharmacists assess bodily parameters and lifestyle habits via a questionnaire. Gyms and sports centers offer an introductory meeting and a free class, and walking groups welcome new participants free of charge. These distinct contributions are supported by a unique branded communication through leaflets, posters, social media contents and a dedicated web-page.

Evaluation is currently ongoing. Campaign will be evaluated based on both stakeholder participation and impact on the target population. Stakeholder participation will be assessed by the number of GPs, pharmacies, sports centres and gyms that take part to the program. Campaign impact on the population will be assessed via changes in physical activity levels and bodily parameter through longitudinal data collected by pharmacists at baseline, 3-month and 6-month intervals.

**Conclusion:**

“Chi si muove, si ama!” campaign represents a multi-stakeholder, cross-sectoral approach to promote physical activity among sedentary adults at regional level. This innovative initiative offers valuable insights for informing policies and practices aimed at improving public health outcomes and promoting population well-being on a broader scale.

